# Heterogeneity in preoperative *Staphylococcus aureus* screening and decolonization strategies among healthcare institutions

**DOI:** 10.1017/ice.2024.231

**Published:** 2025-03

**Authors:** Sarah L. Bennis, Shalini Kulasingam, Patricia Ferrieri, Susan E. Kline

**Affiliations:** 1 Division of Epidemiology & Community Health, School of Public Health, University of Minnesota, Minneapolis, MN, USA; 2 Department of Laboratory Medicine and Pathology, University of Minnesota Medical School, Minneapolis, MN, USA; 3 Division of Infectious Diseases and International Medicine, Department of Medicine, University of Minnesota Medical School, Minneapolis, MN, USA

## Abstract

We surveyed 111 institutions’ practices for screening and decolonization of *Staphylococcus aureus* in presurgical patients. Institutions commonly utilize universal, targeted, or no decolonization strategies. Frequently reported products were nasal mupirocin, chlorhexidine gluconate bathing, and nasal povidone-iodine. Practice variability indicates opportunities to define optimal strategies.

## Introduction

Surgical site infections (SSIs) are a significant problem globally. Annually, over 40 million people in the US undergo surgical procedures, and an estimated 2% of patients will develop an SSI.^1–4^ The most common cause of SSIs, responsible for 30% of cases, is the bacterium *Staphylococcus aureus* (SA).^5^ Approximately 30% of Americans are colonized with SA; SA strains can be either methicillin-susceptible SA (MSSA) or methicillin-resistant SA (MRSA).^5^ Colonization with SA increases the risk of developing an SSI, which can result in lengthier hospitalizations, higher healthcare costs, and death.^2,4,6^

Decolonization of SA carriers before surgery is a potentially effective technique for reducing the risk of SSIs.^7,8^ The last decade has seen the introduction of new decolonization products, such as nasal povidone-iodine and nasal alcohol gel, and more universal presurgical decolonization approaches, compared with the targeted SA screening and decolonization approach. The objective of this study was to sample SA screening and decolonization practices for preoperative patients at a variety of healthcare institutions. We report the results of this survey and compare these findings to our past survey results published in 2012.^9^

## Methods

A survey regarding SA screening and decolonization practices was programmed in REDCap and emailed to members of the Society for Healthcare Epidemiology of America Research Network (SRN), the Minnesota chapter of the Association of Practitioners in Infection Control and Epidemiology (MN-APIC), and the Minnesota Hospital Association (MHA) between May and August 2023. Institutions or their infection prevention team representatives were eligible to participate if they reported membership in at least one of the recruiting organizations and performed any surgery. If institutions submitted more than one survey, we used the most complete survey or the first submission. After completing the survey, participants could enter an optional prize drawing. We report the frequency and percentage of survey responses to summarize current practices for the prevention of SA SSIs.

## Results

One hundred and eighty surveys were initiated with 153 determined to be unique institutions. Response rates were as follows: SRN: 52 of 112 (46.4%), MN-APIC: 40 of 422 (9.5%), and MHA: 53 of 120 (44.2%). Respondents’ institution demographics are presented in Supplemental Table 1. Institutions were from Minnesota (n = 78, 60.0%), 27 other US states (33.8%), and four other countries (n = 4, 6.8%). Most institutions were located in urban communities with >1,000,000 people (n = 29, 24.4%) or small communities with 1,000–10,000 people (n = 27, 22.7%). Most (70%) of the Minnesota institutions were not academic or teaching hospitals and decolonization strategies varied by location (Supplemental Tables 6 and 7). Institutions were commonly described as community hospitals (n = 29, 22.8%), academic medical centers (n = 30, 23.6%), or a combination of more than one category (n = 41, 32.3%). Similar institutional demographics were noted between the 2012 and 2023 surveys (Supplemental Table 2).

**Figure 1.**
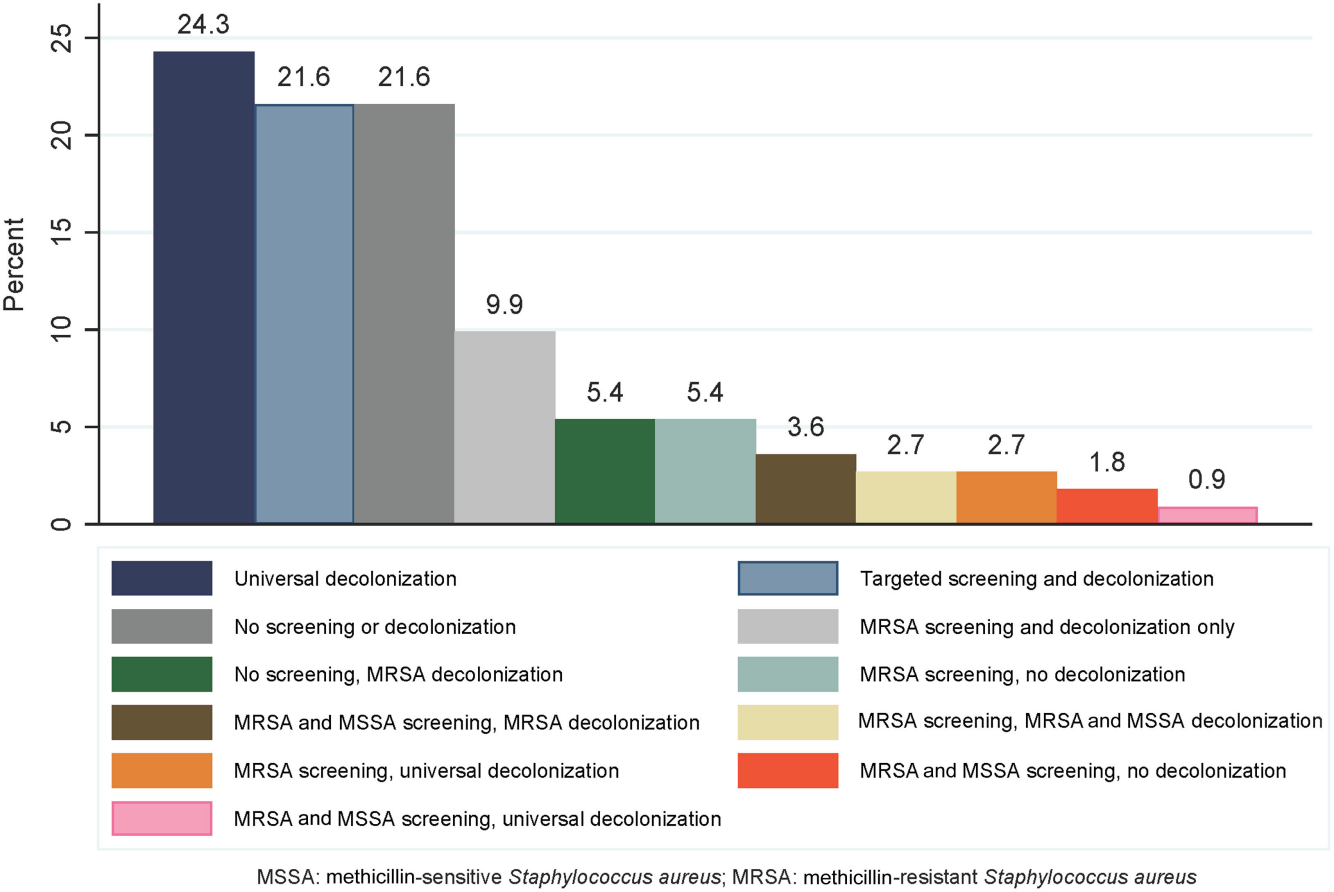
Distribution of screening and decolonization strategies employed by surveyed institutions (N = 111).

One hundred and eleven institutions (72.5%) provided information on their SA screening and decolonization strategies. The most commonly reported strategies were universal decolonization (decolonization of preoperative patients without screening for SA carrier status) (n = 27, 24.3%), no screening or decolonization (n = 24, 21.6%), targeted screening for MSSA and MRSA and decolonization based on carrier status (n = 24, 21.6%), or MRSA-only screening and decolonization (n = 11, 9.9%) (Figure 1).

**Figure 2.**
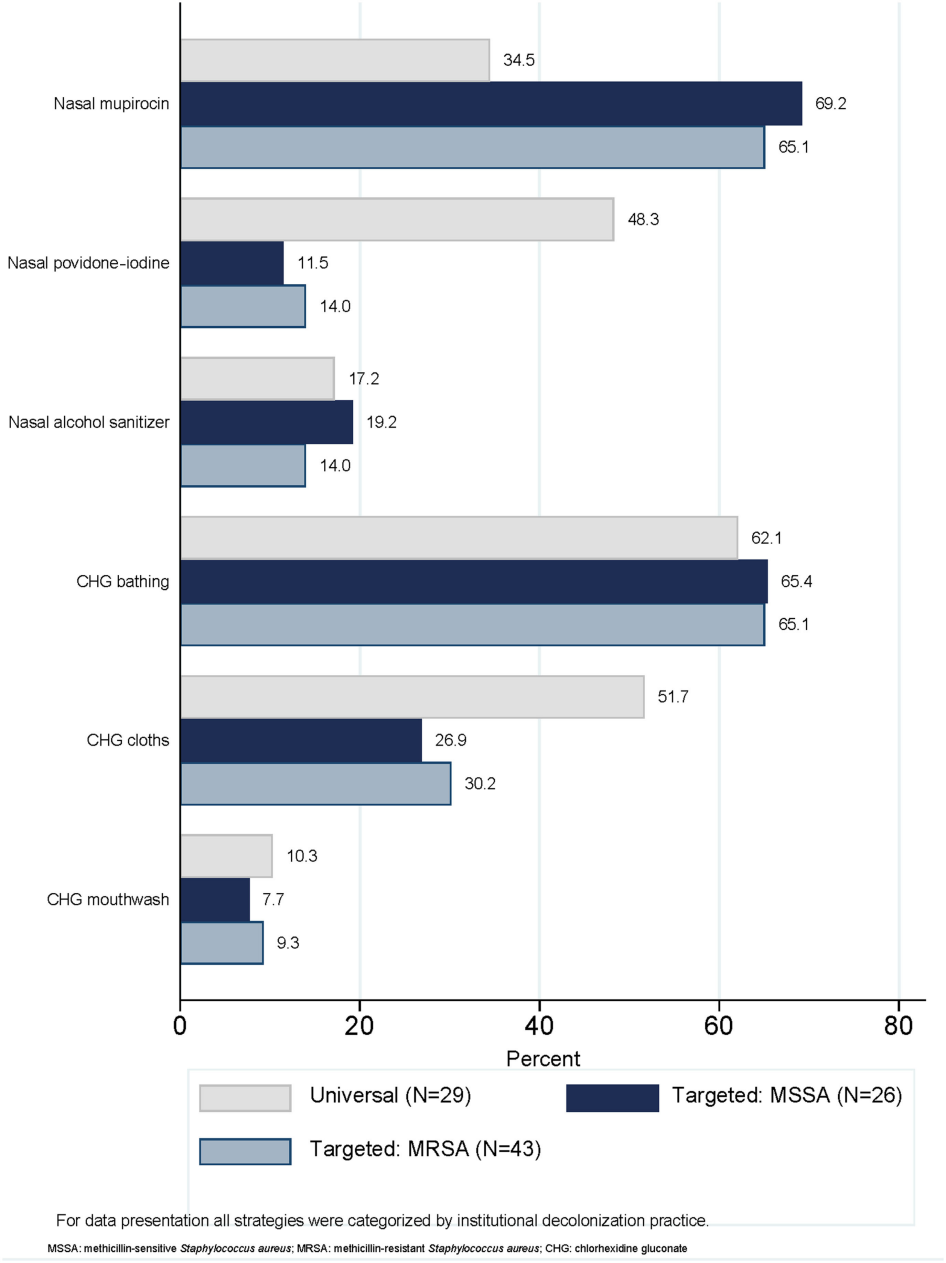
Products used to decolonize preoperative patients by strategy.

Institutions that utilized targeted strategies most frequently reported using nasal mupirocin (n = 18, 69.2%_MSSA_; n=28, 65.1%_MRSA_), chlorhexidine gluconate (CHG) bathing (n = 17, 65.4%_MSSA_; n = 28, 65.1%_MRSA_), and CHG cloths (n = 7, 26.9%_MSSA_; n = 13, 30.2%_MRSA_) (Figure 2). Among the 29 institutions that reported decolonizing products under the universal decolonization strategy, CHG bathing (n = 18, 62.1%), CHG cloths (n = 15, 51.7%), and nasal povidone-iodine (n = 14, 48.3%) were the most prevalent decolonization products. Additionally, a smaller percentage of institutions used nasal alcohol gel (n = 5, 17.2%) for universal decolonization.

Targeted decolonization (MSSA and MRSA) was most frequently used to decolonize patients undergoing cardiovascular (n = 19, 73.1%_MSSA_; n = 22, 50.0%_MRSA_), neurologic (n = 13, 50.0%_MSSA_; n = 17, 38.6%_MRSA_), or orthopedic surgeries (n = 20, 76.9%%_MSSA_; n = 33, 75.0%_MRSA_) (Supplemental Table 3). Universal decolonization was also used most commonly for these three surgery types (Supplemental Table 3). Institutions employing universal decolonization were decolonizing for more surgery types overall (Supplemental Table 3). Institutions not using any screening or decolonization were mostly small hospitals, performed less than 10,000 surgeries annually, or were located in rural communities (Supplemental Table 4).

Nasal mupirocin and CHG bathing were most frequently used for targeted MSSA or MRSA decolonization (Supplemental Table 5). CHG bathing and CHG cloths were most frequently used under the universal decolonization strategy, as well as the combination of CHG bathing and nasal povidone-iodine (Supplemental Table 5).

## Discussion

Compared to the practices found in our survey conducted in 2012, we noted a shift toward universal decolonization and away from targeted SA screening and decolonization. In 2012, 37% of respondents reported targeted screening for SA colonization before surgery, but this decreased to 21% in the present survey.^9^ Universal decolonization, which currently accounts for 24.3% of decolonization, was not reported in the 2012 survey.^9^ A specific question about universal decolonization wasn’t asked then. In 2012, members of the same organizations were surveyed (MN-APIC, MHA, and SRN), with questions focusing on whether routine screening for MSSA and MRSA was done before surgery and whether identified SA carriers were decolonized. In the 2023 survey, we note there is still heterogeneity in practice, reflecting the uncertainty in the optimal decolonization approach and product(s).

Some factors that may have led to the shift away from targeted screening and decolonization for SA carriers are the costs and time of obtaining the cultures or polymerase chain reaction swabs to detect SA carriage and the need to target those individuals with the decolonizing products versus the simplicity of the universal approach with day of surgery application of a nasal product compared with multiple days. Of note, 31.0% of institutions reported the use of CHG soap, cloths, or mouthwash only for universal decolonization but did not add a nasal decolonization product, making it less likely that SA carriage is eradicated.^5^

Future studies may continue to explore the heterogeneity in screening and decolonization practices by considering key unanswered questions, such as whether universal or targeted screening and decolonization are more efficacious and cost-effective. This will inform which strategies are effective while limiting the possibility of resistant bacterial strains due to the widespread use of antiseptic products for non-SA carriers. Future studies may also consider how decolonization of skin and nasal surfaces causes unwanted loss of “healthy” bacteria and how altering microbiome diversity may increase the risk for SSIs.^10^

We note the following limitations of our study. The number of responding institutions is small relative to the number of surveys emailed, with a large proportion from Minnesota. As a result, the strategies reported may not be representative of the practice mix in other US states or international hospitals. The survey was not anonymous, potentially leading to selection bias, but it is unclear how this bias may have influenced the results. We cannot determine the exact combination of products used in institutional decolonization protocols, limiting our reporting of multiple products. Finally, some institutions reported conflicting screening and decolonization practices (eg, no screening and MRSA-only decolonization). For data presentation, all strategies were categorized based on the institutional decolonization practice.

The strengths of our survey include the robust recruitment of verified members of professional organizations with expertise in the field of infection prevention. The survey also asked detailed questions on institutional screening and decolonization practices, allowing us to compare current results to our previous survey in 2012. This report gives a current snapshot in time of the decolonization practices before surgery at a variety of institutions.

## Conclusions

In this survey, universal decolonization emerged as a new strategy employed by institutions to prevent SA SSIs. Many institutions still use targeted decolonization strategies or do not decolonize preoperative patients. The continued heterogeneity in approaches to decolonization may reflect the ongoing uncertainty in optimal SA decolonization practices and emphasize the need for future research to inform evidence-based practices. Additionally, one approach may not be practical or cost-effective for all institutions, depending on the patient population and surgical specialty practiced.

## Supporting information

Bennis et al. supplementary material Bennis et al. supplementary material

## Supplementary material

The supplementary material for this article can be found at https://doi.org/10.1017/ice.2024.231.

## Statement about previous presentation of data/findings

This research was presented in part during a poster session at the SHEA 2024 Spring Meeting in Houston, Texas, April 16–19, 2024.
