# Supplementary material for: Heterogeneity in preoperative Staphylococcus aureus screening and decolonization strategies among healthcare institutions
Source: Infect Control Hosp Epidemiol. Author manuscript; Available in PMC 2025 Mar 7. (PMC11883650; doi:10.1017/ice.2024.231)
Supplement: Supplement [file NIHMS2056846-supplement-Supplement.docx]

| **Supplemental Table 1.** Characteristics of responding institutions. | | |
| --- | --- | --- |
|  | Response Option | N (%) |
| **Recruitment Source** | SHEA Research Network | 52 (34.0) |
|  | MN APIC | 40 (26.1) |
|  | MHA | 53 (34.6) |
|  | Other | 8 (5.2) |
| **Survey-taker job responsibilities** | Infection preventionist | 63 (49.6) |
|  | Infectious disease physician | 8 (6.3) |
|  | Hospital epidemiologist | 10 (7.9) |
|  | Microbiologist | 1 (0.8) |
|  | Nurse | 1 (0.9) |
|  | Other | 1 (0.9) |
|  | More than one job responsibility | 43 (33.9) |
| **State or international location** | Alabama | 1 (0.8) |
|  | Colorado | 2 (1.5) |
|  | Connecticut | 2 (1.5) |
|  | Delaware | 1 (0.8) |
|  | Illinois | 2 (1.5) |
|  | Indiana | 2 (1.5) |
|  | Iowa | 2 (1.5) |
|  | Kentucky | 1 (0.8) |
|  | Maine | 1 (0.8) |
|  | Maryland | 3 (2.3) |
|  | Massachusetts | 3 (2.3) |
|  | Minnesota | 78 (60.0) |
|  | Missouri | 2 (1.5) |
|  | Montana | 1 (0.8) |
|  | Nebraska | 1 (0.8) |
|  | New York | 2 (1.5) |
|  | North Carolina | 1 (0.8) |
|  | North Dakota | 1 (0.8) |
|  | Ohio | 2 (1.5) |
|  | Pennsylvania | 4 (3.1) |
|  | Rhode Island | 0 |
|  | South Carolina | 1 (0.8) |
|  | Tennessee | 1 (0.8) |
|  | Texas | 1 (0.8) |
|  | Vermont | 1 (0.8) |
|  | Washington | 1 (0.8) |
|  | Wisconsin | 4 (3.1) |
|  | Canada | 3 (2.3) |
|  | India | 2 (1.5) |
|  | Malaysia | 2 (1.5) |
|  | South Korea | 1 (0.8) |
| **Institution Type** | Public hospital | 8 (6.3) |
|  | Community hospital | 29 (22.8) |
|  | Teaching hospital | 5 (3.9) |
|  | Government-defined critical access hospital | 6 (4.7) |
|  | Free-standing children's hospital | 1 (0.8) |
|  | Outpatient clinic | 1 (0.8) |
|  | Ambulatory surgical center | 1 (0.8) |
|  | Academic medical center | 30 (23.6) |
|  | Other | 5 (3.9) |
|  | More than one | 41 (32.3) |
| “Other” recruitment source includes: “email” and “infection preventionist”; “Other” job responsibility includes: “Director” | | |

| **Supplemental Table 2.** Brief comparison of responding institution demographics between the 2012 and 2023 surveys. | | |
| --- | --- | --- |
|  | **2012 Survey** | **2023 Survey** |
| N overall | 132 | 111 |
| Number of Responding US States | 26 | 27 |
| Number of Responding Countries | 10 | 5 |
| Proportion from Minnesota | 47.70% | 60.00% |
| Most Commonly Reported Bed Size | 150-299 | >500 |
| Most Commonly Reported Community Size | >1 million | >1 million |
| Most Commonly Reported Institution Descriptions | Community, teaching, and public hospitals | More than one descriptor, academic hospital, community hospital |

**Supplemental Table 3.** Surgery type decolonization stratified by decolonization strategy.

|  | **Decolonization for MSSA (N=26)** | | **Decolonization for MRSA (N=44)** | | **Universal Decolonization (N=29)** | |
| --- | --- | --- | --- | --- | --- | --- |
| **Surgery type** | Yes | No | Yes | No | Yes | No |
|  | n (%) | n (%) | n (%) | n (%) | n (%) | n (%) |
| General | 5 (19.2) | 21 (80.8) | 7 (15.9) | 37 (84.1) | 12 (41.4) | 17 (58.6) |
| Cardiovascular | 19 (73.1) | 7 (26.9) | 22 (50.0) | 22 (50.0) | 13 (44.8) | 16 (55.2) |
| Ear, Nose, Throat | 4 (15.4) | 22 (84.6) | 5 (11.4) | 39 (88.6) | 8 (27.6) | 21 (72.4) |
| Neurology | 13 (50.0) | 13 (50.0) | 17 (38.6) | 27 (61.4) | 13 (44.8) | 16 (55.2) |
| OBGYN | 3 (11.5) | 23 (88.5) | 4 (9.1) | 40 (90.9) | 8 (27.6) | 21 (72.4) |
| Orthopedic | 20 (76.9) | 6 (23.1) | 33 (75.0) | 11 (25.0) | 22 (75.9) | 7 (24.1) |
| Transplant | 2 (7.7) | 24 (92.3) | 3 (6.8) | 41 (93.2) | 7 (24.1) | 22 (75.9) |
| Urology | 3 (11.5) | 23 (88.5) | 4 (9.1) | 44 (90.9) | 8 (27.6) | 21 (72.4) |
| Most surgeries | 3 (11.5) | 23 (88.5) | 9 (20.5) | 35 (79.5) | 8 (27.6) | 21 (72.4) |
| None of the above | 1 (3.9) | 25 (96.1) | 1 (2.3) | 44 (87.7) | 2 (6.9) | 27 (93.1) |
| **MSSA:** methicillin sensitive *Staphylococcus aureus*; **MRSA:** methicillin resistant *Staphylococcus aureus*; **OBGYN:** obstetrics and gynecology  For data presentation all strategies were categorized by their institutional decolonization practice. | | | | | | |

| **Supplemental Table 4.** Institutional screening and decolonization strategies stratified by hospital size, annual surgeries performed, and population size. | | | | | | | |
| --- | --- | --- | --- | --- | --- | --- | --- |
|  | **Hospital size by number of beds (N=98)** | | | **Annual Surgeries Performed (N=111)** | | **Community Population Size (N=111)** | |
| **Screening and decolonization strategy** | Small (0-100), N=29  n (%) | Medium (101-500), N=33  n (%) | Large (>500), N=36  n (%) | ≤10,000 surgeries, N=52  n (%) | >10,000 surgeries, N=59  n (%) | Rural (≤50,000 residents), N=49  n (%) | Urbanized Area (>50,000 residents), N=69  n (%) |
| No screening or decolonization | 11 (37.9) | 7 (21.2) | 3 (8.3) | 18 (34.6) | 6 (10.2) | 18 (42.9) | 6 (8.7) |
| MRSA screening only, MRSA decolonization only | 5 (17.2) | 1 (3.0) | 3 (8.3) | 7 (13.5) | 4 (6.8) | 8 (19.0) | 3 (4.3) |
| MRSA & MSSA screening, MRSA decolonization only | 1 (3.5) | 1 (3.0) | 2 (5.6) | 1 (1.9) | 3 (5.1) | 0 | 4 (5.8) |
| Universal decolonization | 2 (6.9) | 13 (39.4) | 9 (25.0) | 9 (17.3) | 18 (30.5) | 5 (11.9) | 22 (31.9) |
| MRSA & MSSA screening, MRSA & MSSA decolonization | 1 (3.5) | 5 (15.2) | 14 (38.9) | 5 (9.6) | 19 (32.2) | 2 (4.8) | 22 (31.9) |
| Other strategy | 9 (31.0) | 6 (18.2) | 5 (13.9) | 12 (23.1) | 9 (15.2) | 9 (21.4) | 12 (17.4) |
| **MSSA:** methicillin-sensitive *Staphylococcus aureus*; **MRSA**: methicillin-resistant *Staphylococcus aureus*; **Other strategies includes**: MRSA & MSSA screening, no decolonization; MRSA screening only, MRSA/MSSA decolonization; MRSA & MSSA screening, universal decolonization; MRSA screening only, universal decolonization; No screening, MRSA decolonization only; MRSA screening only, no decolonization | | | | | | | |

| **Supplemental Table 5.** Combinations of decolonization products utilized at institutions stratified by decolonization strategy. | | | |
| --- | --- | --- | --- |
|  | Targeted decolonization: MSSA  (N=26) | Targeted decolonization: MRSA (N=43) | Universal decolonization  (N=29) |
|  | n (%) | n (%) | n (%) |
| Nasal mupirocin & CHG bathing | 13 (50.0) | 20 (46.5) | 7 (24.1) |
| Nasal mupirocin & CHG mouthwash | 2 (7.7) | 2 (4.7) | 1 (3.4) |
| Nasal mupirocin & CHG cloths | 5 (19.2) | 9 (20.9) | 6 (20.7) |
| Nasal mupirocin & nasal povidone iodine | 1 (3.8) | 2 (4.7) | 6 (20.7) |
| Nasal mupirocin & nasal alcohol sanitizer | 1 (3.8) | 1 (2.3) | 0 |
| CHG bathing & nasal povidone iodine | 1 (3.8) | 4 (9.3) | 9 (31.0) |
| CHG bathing & nasal alcohol sanitizer | 3 (11.5) | 4 (9.3) | 2 (6.9) |
| CHG bathing & CHG cloths | 2 (7.7) | 8 (18.6) | 12 (41.4) |
| CHG bathing & CHG mouthwash | 2 (7.7) | 4 (9.3) | 3 (10.3) |
| **MSSA:** methicillin sensitive *Staphylococcus aureus*; **MRSA:** methicillin resistant *Staphylococcus aureus;* **CHG:** chlorhexidine gluconate  For data presentation all strategies were categorized by their institutional decolonization practice. | | | |

| **Supplemental Table 6.** Proportion of academic and/or teaching hospital respondents by location. | | | |
| --- | --- | --- | --- |
| **Location** | **Academic or Teaching Hospital** | | |
|  | **Yes  n (%)** | **No  n (%)** | <0.0001* |
| International | 8 (100.0%) | 0 (0%) |  |
| Minnesota | 22 (29.3%) | 53 (70.7%) |  |
| Another US State | 36 (83.7%) | 7 (16.3%) |  |
| *Fisher’s exact chi-square test, α=0.05 | | | |

| **Supplemental Table 7.** Comparison of strategies used in academic and/or teaching hospitals stratified by location. | | | |
| --- | --- | --- | --- |
|  | **Academic and/or Teaching Hospital** | |  |
| **Strategy** | **Yes** | **No** |  |
| ***International Location*** | | |  |
| No screening or decolonization | 4 | 0 |  |
| MRSA screening only, MRSA decolonization | 1 | 0 |  |
| Universal decolonization | 2 | 0 |  |
| MRSA/MSSA screening, MRSA/MSSA decolonization | 1 | 0 |  |
| ***Minnesota*** | | |  |
| No screening or decolonization | 2 | 17 | p-value<0.0001* |
| MRSA screening only, MRSA decolonization | 2 | 4 |  |
| MRSA screening only, no decolonization | 0 | 3 |  |
| MRSA/MSSA screening, MRSA decolonization only | 1 | 2 |  |
| Universal decolonization | 4 | 9 |  |
| MRSA/MSSA screening, MRSA/MSSA decolonization | 8 | 1 |  |
| MRSA/MSSA screening, no decolonization | 1 | 1 |  |
| MRSA screening only, universal decolonization | 2 | 0 |  |
| No screening, MRSA decolonization only | 0 | 5 |  |
| ***Another US State*** | | |  |
| No screening or decolonization | 1 | 0 | p-value=0.658* |
| MRSA screening only, MRSA decolonization | 3 | 1 |  |
| MRSA screening only, no decolonization | 3 | 0 |  |
| MRSA/MSSA screening, MRSA decolonization only | 1 | 0 |  |
| Universal decolonization | 12 | 3 |  |
| MRSA/MSSA screening, MRSA/MSSA decolonization | 12 | 2 |  |
| MRSA screen only, MRSA/MSSA decolonization | 0 | 1 |  |
| No screening, MRSA decolonization only | 1 | 0 |  |
| **MSSA:** methicillin sensitive *Staphylococcus aureus*; **MRSA:** methicillin resistant *Staphylococcus aureus*  *Fisher’s exact chi-square test, α=0.05 | | | |
